# Lateral flow-based assay for simple and rapid visual detection of Asian-type DEL

**DOI:** 10.7717/peerj.21212

**Published:** 2026-04-24

**Authors:** Yotsawat Bunpoom, Chanvit Leelayuwat, Patcharaporn Tippayawat, Molin Wongwattanakul, Piyapong Simtong

**Affiliations:** 1 Biomedical Sciences Program, Graduate School, Khon Kaen University, Khon Kaen, Thailand; 2 Centre for Research and Development of Medical Diagnostic Laboratories, Faculty of Associated Medical Sciences, Khon Kaen University, Khon Kaen, Thailand; 3 Department of Clinical Immunology and Transfusion Sciences, Faculty of Associated Medical Sciences, Khon Kaen University, Khon Kaen, Thailand; 4 Center for Innovation and Standard for Medical Technology and Physical Therapy, Faculty of Associated Medical Sciences, Khon Kaen University, Khon Kaen, Thailand

**Keywords:** Asian-type DEL, RHD 1227A, RHD*01EL.01, D- phenotype, Lateral flow assay

## Abstract

**Background:**

Where a high prevalence of Asian-type DEL (RHD*01EL.01) occurs, simple, rapid, and accurate tests are required to screen patients and donors. Discriminating between Asian-type DEL and RHD*01N.01 (true D-phenotype) is essential to reduce anti-D alloimmunization. This study aimed to develop a simple, and rapid test to detect Asian-type DEL in serologically D- individuals in a Thai population.

**Method:**

In this study, we simplified the performance of the polymerase chain reaction (PCR) combined with a lateral flow assay procedure (PCR-LF), providing a rapid and more sensitive detection of the PCR product and validated it for the identification of Asian-type DEL in samples with a serologically D- phenotype.

**Result:**

In contrast to conventional PCR with agarose gel electrophoresis (PCR-AGE), any PCR products were detected by the lateral flow assay within 10 minutes. This assay accelerates the PCR work schedule and reduces post-PCR detection steps. PCR-LF, PCR-AGE, and Sanger-sequencing assays showed concordant results. The developed PCR-LF assay was more sensitive than an existing PCR-AGE assay for the *RHD 1227A* allele. The estimated time to perform a PCR-LF assay is 2 hours after DNA extraction compared to the 3 hours 30 minutes for PCR-AGE.

**Conclusion:**

PCR-LF was developed to detect Asian-type DEL. This method being rapid and easy to perform would be useful for identifying the Asian-type DEL which serological methods are unable to detect. This could improve blood transfusion management and for the selection of the right blood products to prevent anti-D alloimmunization.

## Introduction

The Rh blood group system (ISBT004) is the most polymorphic and second most clinically important after the ABO blood group system. Currently, 56 Rh blood group antigens are officially registered by the International Society of Blood Transfusion (ISBT). Due to the high immunogenicity of the D antigen, the development of anti-D antibodies can cause life-threatening hemolytic transfusion reactions (HTRs) and hemolytic disease of the fetus and newborn (HDFN) (Turley et al., 2018). Therefore, testing for D antigen is required for all blood donors and patients, to avoid transfusing RhD-positive (D+) red blood cells (RBCs) to RhD-negative (D-) patients. It is of the highest clinical importance to prevent anti-D alloimmunization. The D- phenotype is more common in Caucasians (around 15–17%)(Westhoff, 2007). Total deletion of the *RHD* gene (RHD*01N.01) is the most common cause (>99%) of the D- phenotype in Caucasians. Among Africans, RHDψ (66%), *RHD-CE-D* hybrid gene (15%), and *RHD* gene deletion (18%) are the most prevalent causes of the D- phenotype (Flegel, 2011; Yin, 2023). In East and Southeast Asian populations, D- is rare with a frequency of less than 1%, which often results in a short supply of D- blood in these regions. Importantly, some individuals with a serologically apparent D- phenotype, including Chinese (18%–33%), Korean (13%–18%), Japanese (9%–33%), Thai (10%–33%) and Burmese (17%), are not true D- but exhibit the Del (D-eluate) phenotype (Kwon, Sandler & Flegel, 2017).

The Del phenotype represents a very weak form of D variant (D sites ranging from 20 to 40 molecules/cells) detected only by the anti-D adsorption and elution technique. However, this method is not currently used in routine blood bank practice. Consequently, transfusion from a Del phenotype donor mistyped as the D- phenotype has the potential to elicit anti-D alloimmunization in D- recipients. This risk is supported by clinical evidence: 19 cases of primary and secondary anti-D sensitization, including instances of delayed HTRs following transfusion of Asian-type DEL have been reported among East Asian (Shao et al., 2012; Ito et al., 2021; Suksard et al., 2023). These findings underscore the clinical significance of DEL-associated alloimmunization. To date, 50 DEL alleles have been officially recognized and documented by the ISBT (accessed February 2026) (ISBT Blood Group Database, 2026). Among them, RHD*1227A (RHD*01EL.01), formerly known as *RHD* (K409K) dominates in DEL individuals (>95%) in East and Southeast Asia and is thus termed ‘Asian-type DEL’ (Ji et al., 2023). Genetically, RBCs from Asian-type DEL individuals express the complete repertoire of epitopes as D+ RBCs, contributing to the expression of the D antigen (Körmöczi et al., 2005; Ji et al., 2023). Thus, individuals with Asian-type DEL may safely receive D+ RBC transfusion without risk of alloanti-D immunization and pregnant women do not need Rh immune globulin (RhIG) prophylaxis (Shao, 2010; Shao et al., 2010). In contrast, RBCs from Asian-type DEL individuals can trigger alloanti-D when transfused to recipients who are truly D- (Wagner, 2023; Flegel & Srivastava, 2024).

Currently, polymerase chain reaction (PCR)-based methods are available for the accurate detection of Asian-type DEL, including PCR sequence-specific primers (PCR-SSP), PCR high-resolution melting (HRM) curve analysis, and quantitative multiplex polymerase chain reaction (PCR) of short fluorescent fragments (QMPSF) (Ogasawara et al., 2015; Nuchnoi et al., 2022; Simtong et al., 2022). However, these methods have certain disadvantages. For example, PCR-SSP requires amplicon size analysis involving agarose gel electrophoresis and visual interpretation under UV lamps, which is laborious and time-consuming. Moreover, highly sensitive amplification for the HRM and QMPSF methods requires special equipment and trained personnel, making them difficult and impractical as a point-of-care test (POCT). Therefore, a simple and rapid genotyping technique is essential. These drawbacks of molecular genotyping methods can be overcome by providing a rapid POCT for nucleic acid detection. However, the current POCTs for specific nucleic acid detection still requires three main steps (i) sample preparation, (ii) amplification, and (iii) amplicon detection (Niemz, Ferguson & Boyle, 2011).

PCR combined with lateral flow (PCR-LF) assays are currently used for the diagnosis of many infectious diseases (Najomtien et al., 2024). PCR-LF is simple and faster than conventional PCR with agarose gel electrophoresis (PCR-AGE). The PCR-LF assay utilizes immunochromatographic principles to detect dual-labeled amplicons. PCR products labeled with fluorescein isothiocyanate (FITC) and Biotin migrate along a nitrocellulose membrane. FITC-labeled amplicons bind to anti-FITC-conjugated gold nanoparticles, while the Biotin label is captured by immobilized streptavidin at the test line, resulting in a visible red-purple band. A control line ensures assay validity by capturing excess gold conjugates. This method uses less time and equipment than gel electrophoresis, and it reduces the post-PCR processing steps. Thus, it would be more cost-effective for the diagnosis of Asian-type DEL in regions with a high prevalence. Therefore, this study aimed to develop a detection system based on PCR-LF for rapid and easy detection of Asian-type DEL, the most frequent *RHD* allele found in serologically D- individuals in East and Southeast Asian countries.

## Materials & Methods

### Samples

Ethical approval for this study was obtained from the Institutional Review Board of Khon Kaen University, Thailand (HE671487). This study, used leftover specimen covered by a previous broad consent form: no additional consent was required. A total of 40 anonymized genomic DNA samples with known RHD*1227A status were used for this study; 20 samples were RHD*01N.01 and the remaining 20 samples had the Asian-type DEL allele. All samples had been typed for RHD*1227A by PCR-SSP, as reported in a previous study (Pittayabumrung et al., 2025). In addition, 20 samples with Asian-type DEL were confirmed and further reference genotyping for assay validation using Sanger sequencing. The DNA concentration and purity were assessed using a Nanodrop spectrophotometer (Dynamica Scientific, Dietikon, Switzerland). We used the STARD checklist when writing our report (Bossuyt et al., 2015).

### Molecular detection of Asian-type DEL

#### PCR with agarose gel electrophoresis (PCR-AGE)

The sequences of forward and reverse primers used for the PCR amplification of the Asian-type DEL allele were similar to those previously reported (Yang et al., 2007) with minor modification. For the PCR-AGE assay, the specific forward primer was labeled at its 5′ end with FITC (5′-FITC-GATGACCAAGTTTTCTGGAAA-3′) for antigenic detection. The reverse primer was labeled with biotinylated (5′-biotin-TCTGTCACCCGCATGTCAG-3′) to enable lateral flow (LF) detection. Both primers were synthesized by the Synbio-Technologies company, USA. To ensure specificity, these primers were analyzed using a nucleotide primer-Basic Local Alignment Search Tool (BLAST) search available through the National Center for Biotechnology Information (NCBI) website. A 346-bp PCR product is expected for Asian-type DEL allele.

PCR reactions were carried out in a 13 µL final volume, consisting of 1X PCR buffer (67 mM Tris–HCl, pH 8.8; 17 mM ammonium sulfate; 0.1% Tween-20), 0.72 mM dNTPs (Vivantis, Kuala Lumpur, Malaysia), 3.25 mM MgCl_2_, 5 U of Taq DNA polymerase (Invitrogen, Madison, WI, USA), 3% DMSO,12.5 ng/µL of genomic DNA, and forward and reverse primers at a final concentration of 0.1 µM. PCR amplification was performed using an Applied Biosystems Veriti™ Thermal Cycler (Life Technologies, Foster City, CA, USA). The cycling conditions included an initial denaturation at 94 °C for 2 min, followed by five cycles of denaturation at 96 °C for 30 s, annealing at 65 °C for 60 s, and extension at 72 °C for 40 s. This was followed by 21 cycles of denaturation at 96 °C for 30 s, annealing at 60 °C for 1 min, and extension at 72 °C for 40 s. A final set of four cycles was performed with denaturation at 96 °C for 30 s, annealing at 55 °C for 1 min and 15 s, and extension at 72 °C for 2 min. A final extension step was carried out at 72 °C for 10 min, followed by an indefinite hold at 4 °C. PCR amplicons were resolved by electrophoresis on a 1.5% agarose gel in 0.5X Tris-borate-EDTA (TBE) buffer. Gels were stained with RedSafe™ nucleic acid staining solution (iNtRON Biotechnology, Inc., Seongnam, Gyeonggi-do, South Korea) and visualized under UV illumination using a Syngene gel documentation system (Syngene, Maryland, USA).

#### PCR combined with lateral flow (PCR-LF)

**Figure 1:**
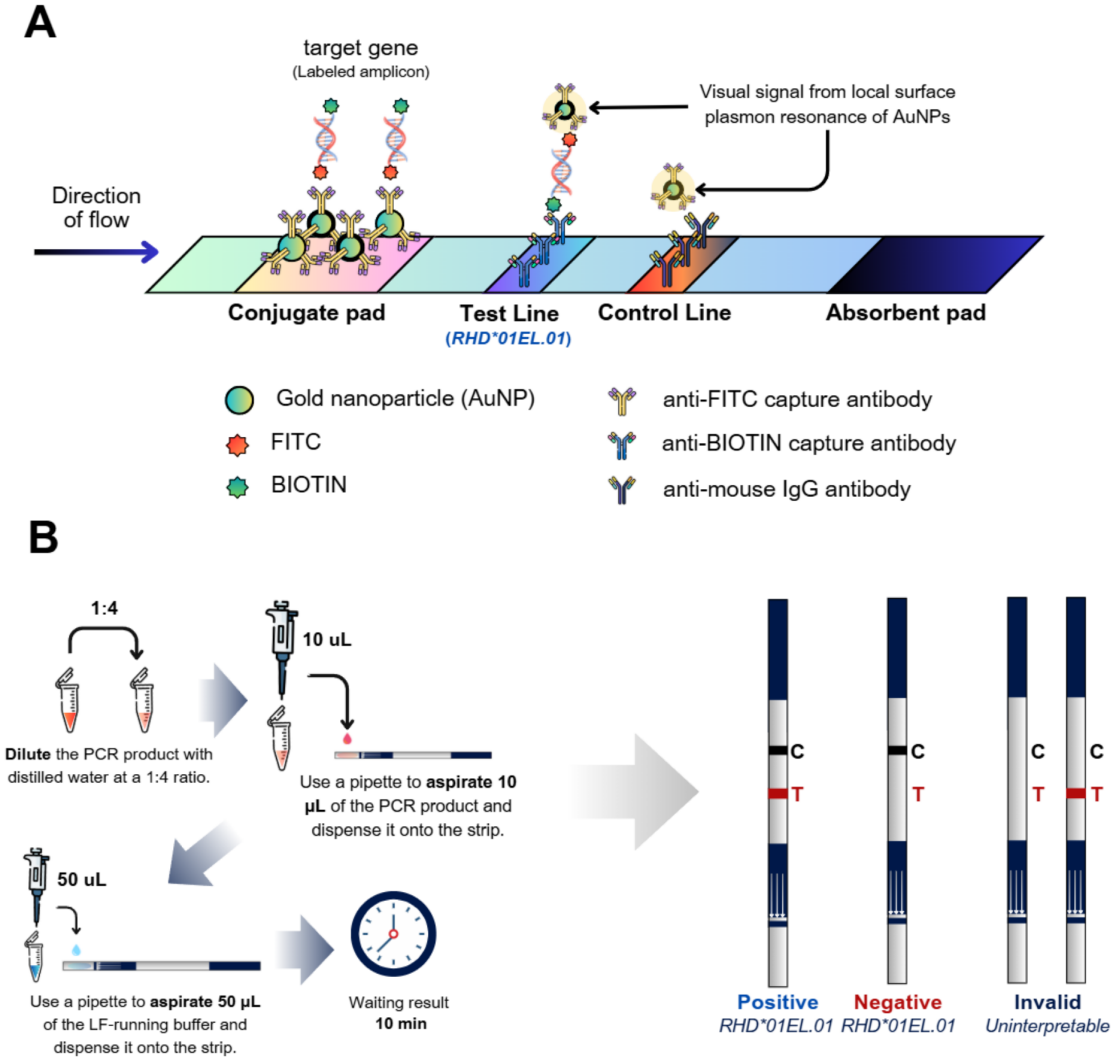
Schematic diagram of the PCR-LF assay. (A) (i) The forward and reverse primers generate biotin- and FITC-labeled PCR products. (ii) The FITC-labeled amplicon binds to the anti-FITC capture antibody coated on gold nanoparticles (AuNPs). (iii) The biotin-labeled amplicon binds to the anti-biotin capture antibody immobilized on the test line. (iv) Excess AuNP-conjugated anti-FITC antibodies are captured by the anti-mouse IgG antibody coated on the control line. (v) The presence of two visual bands (test and control lines) indicates a positive result, whereas a single band at the control line indicates a negative result. (B) Schematic workflow of the PCR-LF assay. (i) Dilute the PCR product with distilled water with 1:4 ratio. (ii) Carefully pipette 10 µL of the PCR product onto the sample pad. (iii) Pipette 50 µL of running buffer onto the sample pad. (iv) Wait approximately 10 min for the results. A positive result indicates the presence of the RHD*01EL.01 allele, whereas a negative result confirms its absence. Figure generated by Canva [Graphic design software].

For the PCR-LF assay, PCR amplification was performed using the conditions mentioned above but using 0.025 µM of each primer. The PCR products were subsequently analyzed using strip chromatography (Kestrel Bioscience, Co., Ltd., Thailand) (Fig. 1A).

For PCR-LF analysis, PCR products were diluted at a 1:4 ratio with distilled water. A total of 10 µL of the diluted sample was applied onto the sample pad. Subsequently, 50 µL of LF-running buffer (Kestrel Bioscience, Co., Ltd., Thailand) was added to the same pad, and the reaction was allowed to proceed at room temperature for approximately 10 min. Results were interpreted by the naked eye based on the visible presence of test and control lines: a positive result was indicated by the appearance of two lines (test and control), while a negative result was determined by the presence of only the control line. The control line served as an internal quality control to confirm the proper functionality of the lateral flow strip (Fig. 1B).

In addition, the intensity of the test and control lines was quantitatively assessed using the RapidScan ST5 reader (Eurofins Technologies, California, USA) to ensure objective and reproducible results.

### Sanger sequencing

To confirm the presence of the Asian-type DEL allele, twenty samples positive for *c.1227G>A* in *RHD* exon 9 were sequenced using Sanger sequencing and compared with reference sequence (NCBI Gene ID: NG_007494.1). Sequencing was performed by Bio Basic Inc. (Canada) using fluorescent dye-terminator sequencing on an ABI Prism™ 3730x DNA sequence. The sequencing reaction utilized the same forward and reverse primers as described in the PCR-AGE method. Sequence data were analyzed using SnackVar V2.4.3 software for variant identification.

### Detection of limits of the PCR-AGE and PCR-LF

To assess the sensitivity of PCR-LF for detecting the Asian-type DEL allele, a two-fold serial dilution of genomic DNA was prepared at concentrations of 12.5 ng/uL, 6.25 ng/uL, 3.12 ng/uL, 1.56 ng/uL, and 0.78 ng/uL. These dilutions were used as templates for PCR amplification, with a primer concentration of 0.025 µM/µL. Following amplification, PCR products were diluted at a 1:4 ratio and applied to lateral flow strips for visualization. The intensity of the test and control lines was measured and compared across different DNA input concentrations. For comparison, PCR-AGE was performed using the same DNA and primer concentrations. PCR reactions were conducted under identical cycling conditions, but the undiluted PCR products were analyzed *via* agarose gel electrophoresis to assess amplification efficiency and product integrity.

### Statistical analysis

Statistical analyses were performed using GraphPad Prism version 10.1.2 (GraphPad Software, USA). Signal intensities from the lateral flow assay were expressed as mean ± standard deviation (SD). The cut-off value for positive detection was established based on the mean signal intensity of negative control samples plus three times the standard deviation (mean + 3SD), providing a stringent threshold to distinguish positive from negative results. Sensitivity was calculated as the number of true positives (TP) divided by the sum of true positives and false negatives (TP/(TP + FN)), and specificity was calculated as the number of true negatives (TN) divided by the sum of true negatives and false positives (TN/(TN + FP)), using Sanger DNA sequencing as the reference method.

## Results

### Comparison between PCR-AGE and PCR-LF procedure

The PCR-AGE assay was initially used to detect the Asian-type DEL allele. Following PCR amplification, the amplicons were analyzed by agarose gel electrophoresis and visualized under UV light. The PCR-LF assay, performed under the same PCR conditions including identical thermal cycling profiles, PCR-buffer components, specific primers, and DNA template concentrations also successfully detected the target amplicons. The products were diluted 1:4 with distilled water and applied directly to the sample pad of a lateral flow strip. Signal intensities were recorded within approximately 10 min. (Table 1).

### Specificity of the PCR-LF

**Figure 2:**
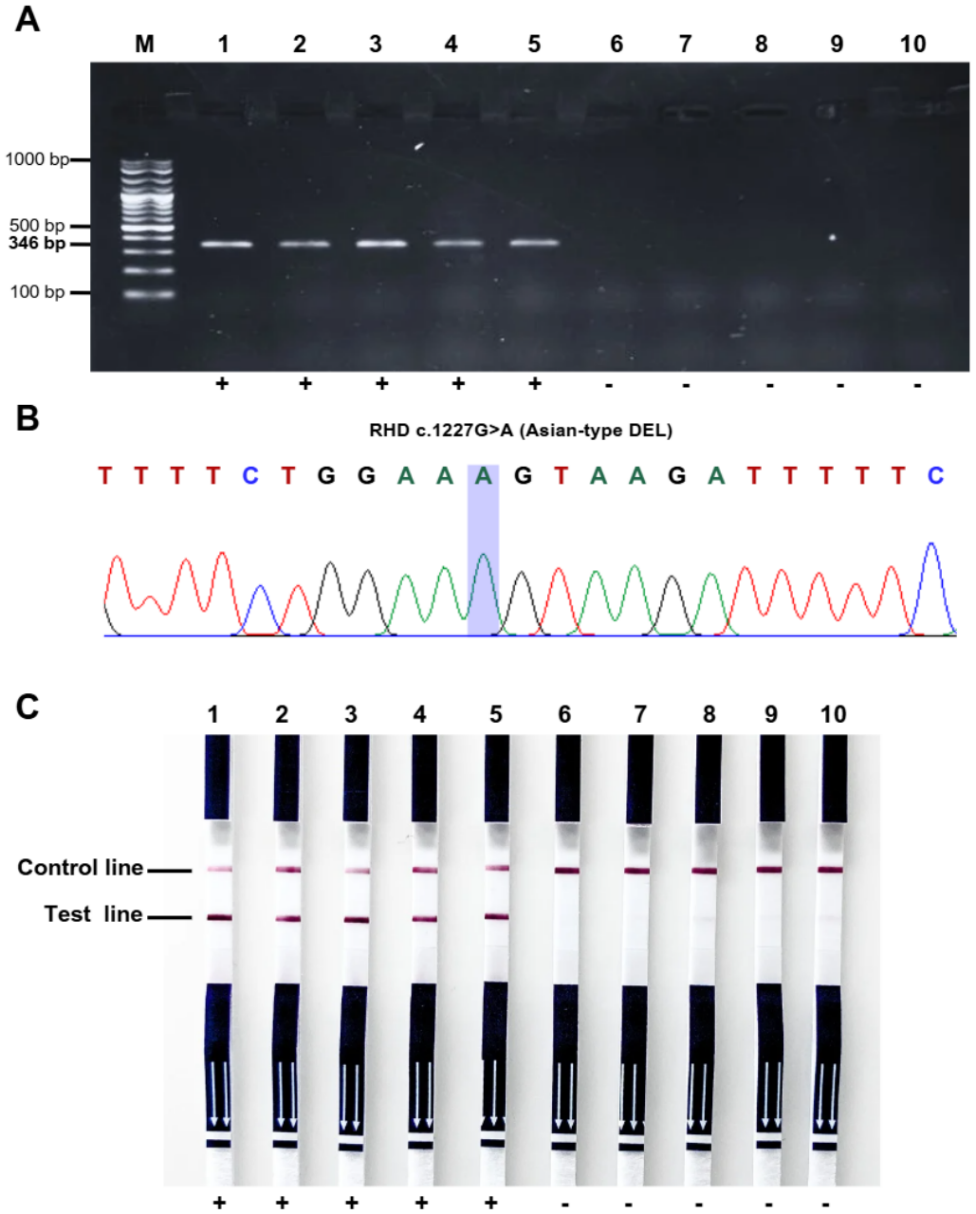
Detection of RHD*01EL.01 allele using three different assays. (A) Representative gel showing RHD*01EL.01 typing by PCR-AGE. Lane M: A 100 bp plus DNA ladder, NL1407, Vivantis. The 346 bp amplified product of RHD*1227A allele as a genetic marker for Asian-type DEL is present in samples: RH105 (1), RH107 (2), RH108 (3), RH120 (4) and RH203 (5) and were not amplified in samples RH13 (6), RH17 (7), RH63 (8), RH61.2 (9), and RH63 (10) as RHD*01N01. (B) Sanger sequencing. A representative sequencing pattern of RHD*01EL.01, the blue marker indicates the position of the RHD c.1227G>A substitution in RHD exon 9. (C) PCR-LF assay to the following reference DNA samples: RH105 (1), RH107 (2), RH108 (3), RH120 (4), RH203 (5), RH13 (6), RH17 (7), RH63 (8), RH61.2 (9), and RH63 (10).

To evaluate the specificity of the PCR-LF assay. Firstly, 10 genomic DNA samples were analyzed for the *RHD1227A* allele as a genetic marker for Asian-type DEL using PCR-AGE and Sanger sequencing. The results identified five samples as Asian-type DEL and five as RHD*01N.01 (Figs. 2A, 2B). Following sample characterization, all 10 samples were tested using PCR-LF assay. The assay successfully all five Asian-type DEL samples as positive, while all five RHD*01N.01 samples tested negative (Fig. 2C). These findings were consistent with the results obtained from PCR-AGE and Sanger sequencing, demonstrating the comparable specificity of the PCR-LF assay in distinguishing Asian-type DEL from RHD*01N.01 alleles.

**Table 1:** Comparison of PCR-AGE and PCR-LF assay.

**Assays**	**PCR-AGE**	**PCR-LF**
DNA Concentration	12.5 ng/µL	12.5 ng/µL
Primer Concentration	0.1 µmol	0.025 µmol
Primer Design	FITC and Biotin labeled	FITC and Biotin labeled
Post-PCR Processing	– Undiluted amplicon – Agarose gel preparation: 30 min – Electrophoresis: 25 min – UV visualization: 5 min	– 1:4 diluted amplicon – After adding PCR product and running buffer: 10 min waiting time
Limit of Detection (LoD)	– DNA: 12.5 ng – Primer: 0.05 µM	– DNA: 3.12 ng – Primer: 0.025 µM
Turnaround Time	3 h 30 min	2 hours
Cost Breakdown	– DNA extraction: $5.94 USD – Reagents & consumables: $7USD – Agarose gel: $2.97 USD Total: $16.00 USD	– DNA extraction: $5.94 USD – Reagents & consumables: $7 USD – PCR buffer & strip: $3.27 USD Total: $16.21USD

**Figure 3:**
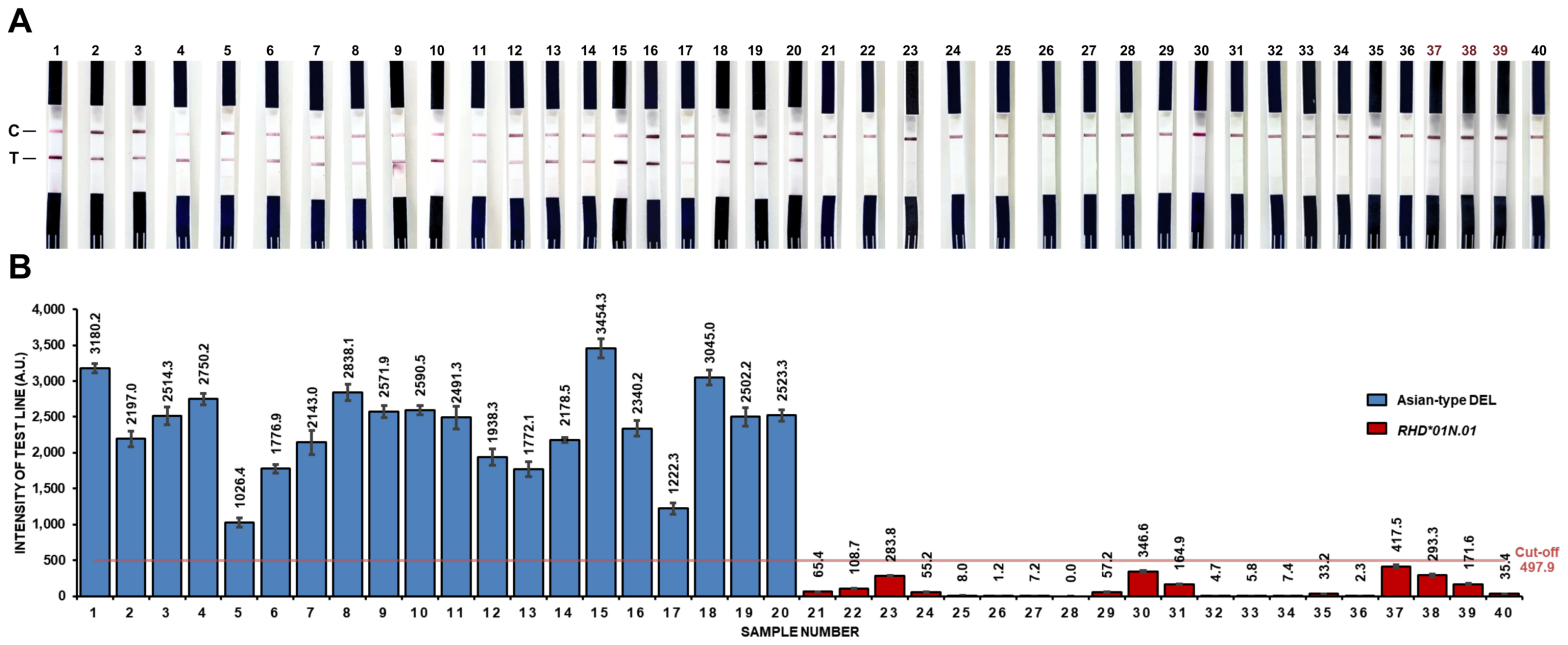
Test performance evaluation of the PCR-LF assay. (A) Representative test strip results from 20 Asian-type DEL samples (1–20) and 20 RHD*01N01 samples (21–40). The visual interpretation corresponds with intensity-based classification. (B) Distribution of test line intensity values for all 40 samples, including 20 Asian-type DEL, (samples 1–20) and 20 RHD*01N01 (samples 21–40). The cutoff line (red line) determined using the mean + 3SD method was 497.9 A.U. Data are presented as mean ± SD of triplicate test.

The PCR-LF assay was then applied to test 40 samples known to be either negative or positive for the Asian-type DEL allele. The test correctly reported all 20 samples with the Asian-type DEL marker as positive and 17 out of 20 samples without the marker as negative (Fig. 3A). In the three false positives, faint bands were detectable with the naked eye. Based on this, the PCR-LF assay achieved a sensitivity of 100% and specificity of 85%. While these data underscore the high sensitivity of the assay, they also indicate the need for additional refinement, particularly relating to the definition of the band intensity threshold to reduce false positives and enhance specificity.

### Determination of cutoff value for Asian-type DEL detection

Intensity measurements for known Asian-type DEL (*n* = 20) and RHD*01N.01 (*n* = 20) samples were used to establish the optimal cutoff value for Asian-type DEL detection using the PCR-LF assay (Figs. 3A, 3B). A statistical method based on mean ± 3 SD was used to define the cutoff. The calculated cutoff value obtained using this method was 497.9 Arbitrary Units (A.U.). Samples with intensity ≥ 497.9 A.U. were classified as Asian-type DEL (Table 2).

### Limit of detection of PCR-LF and PCR-AGE

**Figure 4:**
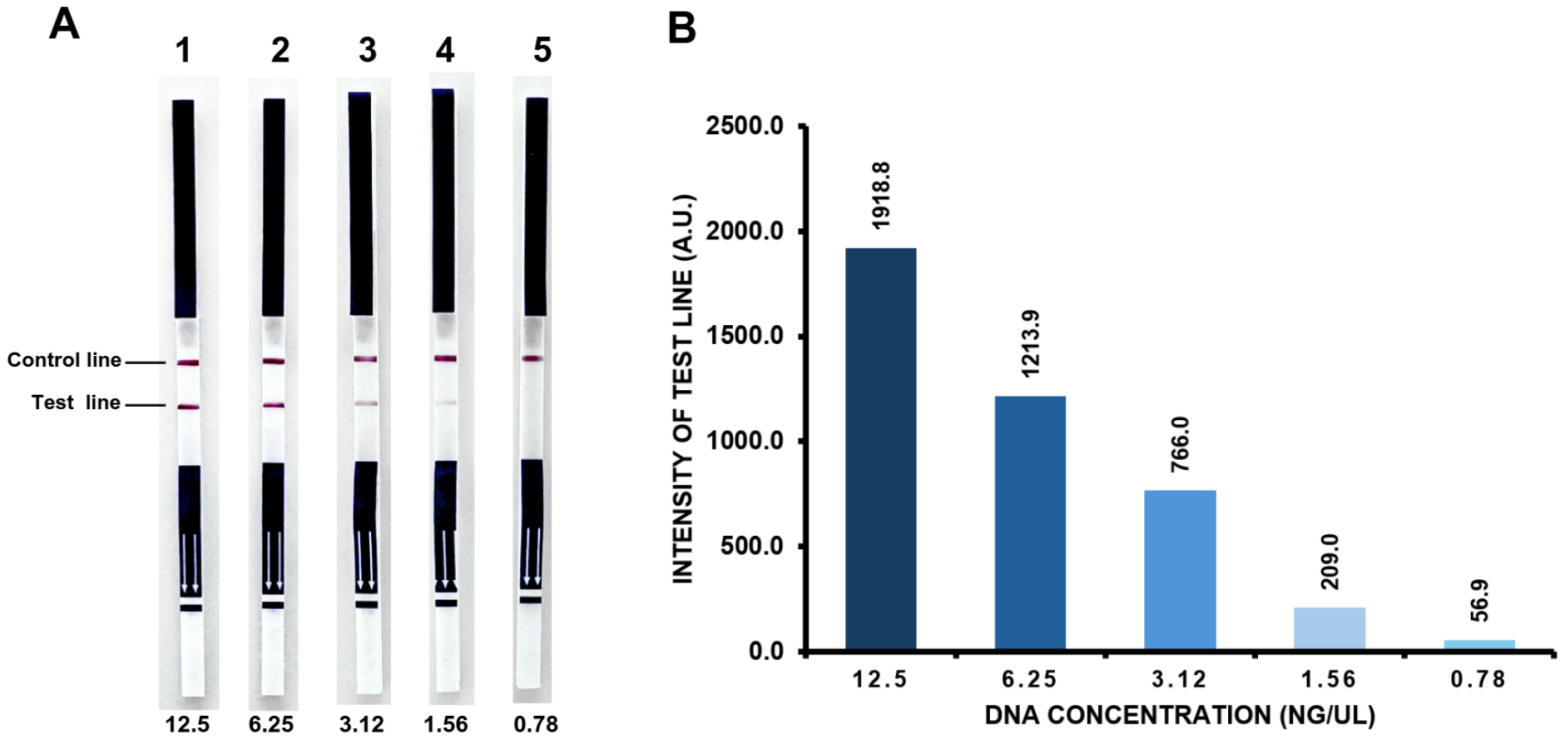
Limit of detection. (A) Representative lateral flow strip results and (B) corresponding intensity measurements. The result demonstrating the assay’s detection sensitivity across varying DNA concentrations (RH107): (1) 12.5 ng/µL, (2) 6.25 ng/µL, (3) 3.12 ng/µL, (4) 1.56 ng/µL, and (5) 0.78 ng/µL. The results establish the assay’s LoD by determining the lowest DNA concentration at which a detectable signal is observed. This finding highlights the assay’s potential for low-template DNA analysis and its applicability in sensitive molecular diagnostics.

The results from the lateral-flow strip assay demonstrate a correlation between DNA template concentration and signal intensity. The measured intensities at varying DNA concentrations are as follows: 12.5 ng/µL–1,918.8 A.U., 6.25 ng/µL–1,213.9 A.U., 3.12 ng/µL–766 A.U., 1.56 ng/µL–209 A.U., and 0.78 ng/µL–56.9 A.U.. Based on a defined cut-off value of 497.9 A.U. for positive detection (Fig. 3A), concentrations of ≥ 3.12 ng/µL were considered positive, while < 3.12 ng/µL are considered negative. Visual inspection also confirmed the fading and eventual disappearance of the test line at lower concentrations. Therefore, under the current assay conditions and cut-off criteria, the limit of detection (LoD) for the PCR-LF assay is determined to be 3.12 ng/µL. (Fig. 4). Additionally, PCR products analyzed by agarose gel electrophoresis (PCR-AGE) under the same amplification conditions showed no visible bands at any DNA concentration, indicating that the product levels were below the detection threshold of gel-based analysis.

**Table 2:** Comparison of test line intensities between Asian-type DEL and RHD*01N.01 samples.

**Sample group**	**Number of samples (n)**	**Mean intensity (A.U.)**	**Range (Min–Max)**	**Interpretation**
Asian-type DEL	20	2,347.5	1,026.4–3,454.3	Positive (≥ 497.9 A.U.)
RHD*01N.01	20	135.2	0.0–417.5	Negative (<497.9 A.U.)

## Discussion

Identifying the Asian-type DEL allele in individuals with a serologically D- phenotype is critical for precision in transfusion medicine. PCR-based approaches remain the gold standard among various molecular methods due to their reliability and accuracy (Buchta et al., 2020). PCR-AGE is widely employed for Asian-type DEL detection (Ito et al., 2021; Simtong et al., 2022; Suksard et al., 2023). Its workflow requires approximately 3 h 30 min and needs a UV transilluminator to visualize PCR products. To streamline the detection process, a more rapid and user-friendly method is required to replace gel electrophoresis.

In this study, we developed a PCR-LF assay, integrating PCR-SSP with lateral flow-strip detection, to enable efficient identification of Asian-type DEL alleles in serologically D- individuals. This approach allows amplicons to be analyzed using a lateral flow strip, eliminating the need for gel electrophoresis. The workflow consists of sample loading onto the strip in the presence of LF-running buffer, with naked-eye visual interpretation of results occurring within 10 min. This design offers key advantages, including reduced processing time, simplified workflow, and elimination of specialized electrophoresis equipment. Our findings demonstrated that PCR-LF exhibits high concordance in detecting the Asian-type DEL allele. Critical parameters influencing assay performance include primer concentration, reagent composition, PCR cycling conditions, and reagent volume. Optimization of strip reagent composition and visualization time is essential for reliable result interpretation. Most notably, the PCR-LF assay demonstrated superior analytical sensitivity, with a LoD of 3.12 ng, which is 4-fold more sensitive than the conventional PCR-AGE (LoD of 12.5 ng). Coupled with a substantial 43% reduction in turnaround time, these findings clearly highlight the technical advantages and practical efficiency of our method over the standard agarose gel-based detection.

Despite its advantages, the PCR-LF assay still needs a PCR thermal cycler. Since the COVID-19 pandemic, hospitals often have easy access to thermal cyclers and DNA extraction systems, making this approach feasible for routine application in blood banks.

Currently, recombinase polymerase amplification (RPA) is one of the few amplification techniques that is considered an isothermal approach because it does not require a thermal cycler. Detection of RPA amplicons can be done using agarose gel electrophoresis, lateral flow strips (Körmöczi et al., 2005; Li, Macdonald & Von Stetten, 2018; McQuillan & Wilson, 2021; Srisrattakarn et al., 2022), or even with bio-sensors, but there is currently only one supplier of the reaction kit which limits the commercial accessibility of this method.

The use of lateral flow strips and running buffer in PCR-LF assays contributes to higher costs compared to PCR-AGE methods, with the strip alone costing approximately $3.27 per test. Additionally, the use of FITC-labeled primers further increases expenses due to their higher synthesis cost and limited stability. Replacing FITC with digoxigenin (DIG), a more stable and cost-effective labeling alternative, could significantly reduce assay costs and enhance the feasibility of PCR-LF for routine application, particularly in low-resource settings (Soliman, Kumar & El-Matbouli, 2018; Srisrattakarn et al., 2022).

The Asian-type DEL allele is most common among Asian populations. According to a study including 1,270 D- samples from Thailand, the frequency of Asian-type DEL allele was 7.62% with frequency of hybrid allele RHD*01N.03 (3.46%) and RHD*01EL.44 (0.08%) (Nuchnoi et al., 2022). *RHD* genotype *RHD* (M295I) (c.884G>A exon 6) is more prevalent in European and mixed populations, and *RHD* (IVS3+1G>A) is found in Indian and Southeast Asian populations, This indicates the diversity of genetic bases of RhD variants (Flegel, 2007; Gu et al., 2014). Our study focused specifically on the DEL allele of Asian type. We designed the primers to amplify exon 9 (c.1227G>A) that was specifically present in the Asian-type DEL allele. Therefore, the amplification of other DEL variant alleles was unlikely. To precisely determine the RhD phenotype and minimize the potential for misclassification, comprehensive molecular screening is necessary, which is crucial for transfusion compatibility in diverse ethnic populations. Additional DEL variants should be included for the broader applicability of RhD genotyping worldwide.

In Thailand, approximately 15% of serologically D- individuals carry the Asian-type DEL. While only a few cases of anti-D alloimmunization have been reported, the clinical consequences particularly for D- women of childbearing age can be severe, potentially leading to HDFN and delayed HTRs. Thus, the implementation of the PCR-LF assay is justified by its clinical utility and the fact that its cost per test is only marginally higher than conventional PCR methods (Table 1). According to a nation-wide investigation of Thai blood donors, 98.3% of Asian-type DEL carriers were found to be C and/or E antigen-positive, with C being the more common. This strong association facilitates a highly efficient selective screening strategy; by prioritizing D- individuals who are C-positive for PCR-LF testing, clinical laboratories can significantly reduce the molecular testing workload while maintaining high diagnostic sensitivity. In cases with a ccee phenotype and negative PCR-LF results, antigen typing alone can effectively guide further testing protocols (Thongbut et al., 2021; Nuchnoi et al., 2022). Identifying the Asian-type DEL is also crucial for optimizing blood resource management. Since Asian-type DEL individuals can be treated as D+, identifying this allele ensures that limited D- blood units are reserved for ‘true’ D-recipients. Furthermore, recognizing this allele in D- mothers with D+ newborns can reduce the unnecessary administration of Rh immunoglobulin (RhIG) (Koelewijn et al., 2008). Given the increasing proportion of Asian ethnic populations worldwide with the Asian-type DEL allele these results underscore a growing global necessity for standardized molecular screening strategies (Flegel & Srivastava, 2024).

A limitation of this study is that the PCR-LF assay has not yet been validated on a sufficiently large and diverse clinical sample set. To robustly establish diagnostic performance, including sensitivity and specificity, it is generally recommended to test at least 73 positive samples, calculated using the formula n = [Z^2^×p×(1-p)]/d^2^ where *Z* = 1.96 (95% confidence), *p* = 0.95 (expected sensitivity), and *d* = 0.05 (precision), to achieve a ±5% margin of error. A similar number of negative samples is recommended for specific assessment. Future validation should include these sample sizes to ensure reliable test performance.

## Conclusions

PCR-LF assay is a rapid, simple, and near point-of-care genetic test for efficient identification of Asian-type DEL without complex laboratory processes. More work is needed to validate results in a diverse population at the clinical level, optimize parameters of nucleic acid assays, and implement enrichment strategies for molecular screening in the blood bank setting. Widening these strategies will increase transfusion safety and aid international advancements in precision medicine.

##  Supplemental Information

10.7717/peerj.21212/supp-1 Supplemental Information 1 Triplicate intensity of PCR-LF The raw data shows all triplicate intensity of 40 samples.

10.7717/peerj.21212/supp-2 Supplemental Information 2 STARD checklist

## Additional Information and Declarations

### Data Availability

The following information was supplied regarding data availability: The raw data of intensity measurements in the Supplemental File.
